# Arbuscular mycorrhizal fungi regulate soil respiration and its response to precipitation change in a semiarid steppe

**DOI:** 10.1038/srep19990

**Published:** 2016-01-28

**Authors:** Bingwei Zhang, Shan Li, Shiping Chen, Tingting Ren, Zhiqiang Yang, Hanlin Zhao, Yu Liang, Xingguo Han

**Affiliations:** 1 State Key Laboratory of Vegetation and Environmental Change, Institute of Botany, Chinese Academy of Sciences, Beijing, 100093, China; 2 University of Chinese Academy of Sciences, Beijing, 100049, China; 3 State Key Laboratory of Forest and Soil Ecology, Institute of Applied Ecology, Chinese Academy of Sciences, Shenyang 110164, China

## Abstract

Arbuscular mycorrhizal fungi (AMF) are critical links in plant–soil continuum and play a critical role in soil carbon cycles. Soil respiration, one of the largest carbon fluxes in global carbon cycle, is sensitive to precipitation change in semiarid ecosystems. In this study, a field experiment with fungicide application and water addition was conducted during 2010–2013 in a semiarid steppe in Inner Mongolia, China, and soil respiration was continuously measured to investigate the influences of AMF on soil respiration under different precipitation regimes. Results showed that soil respiration was promoted by water addition treatment especially during drought seasons, which induced a nonlinear response of soil respiration to precipitation change. Fungicide application suppressed AMF root colonization without impacts on soil microbes. AMF suppression treatment accelerated soil respiration with 2.7, 28.5 and 37.6 g C m^−2^ across three seasons, which were mainly caused by the enhanced heterotrophic component. A steeper response of soil respiration rate to precipitation was found under fungicide application treatments, suggesting a greater dampening effect of AMF on soil carbon release as water availability increased. Our study highlighted the importance of AMF on soil carbon stabilization and sequestration in semiarid steppe ecosystems especially during wet seasons.

Plant roots may host many fungal species including mycorrhizal fungi, endophytes and pathogens^1^. More than 80% of terrestrial plant species form associations with arbuscular mycorrhizal fungi (AMF) as the symbiotic mycorrhiza^2^, which provide critical links in the plant–soil continuum^3^. It has been reported that AMF play important roles in supplying nutrients to host plants^4^,^5^ as well as determining and maintaining plant community structure and diversity^6^,^7^.

The impacts of AMF on soil carbon cycling are also receiving more and more attentions recently. Firstly, AMF are important components of soil microbial community and contribute to soil respiration. It has been reported that these fungi and their extraradical mycorrhizal hyphae (EMH) constitute as much as 20~30% of soil microbial biomass^8^; and their contribution to total soil respiration ranges from 6% to 25%^9^,^10^,^11^. Secondly, EMH can transfer carbon away from the rhizosphere soil with high respiratory activity, because they consist of a large and complex hyphae network in the soil. This property facilitates soil carbon sequestration^12^,^13^. Thirdly, AMF are important sources and components of the soil organic carbon (SOC) pool^12^,^14^. The fast turnover rate of their EMH leaves large amount of chitin^15^ and glomalin-related soil protein^16^ in the soil, both of which are resistant to decomposition^17^,^18^,^19^. Lastly, by entangling, enmeshing and binding, fungal hyphae facilitate the formation and stabilization of soil aggregates, especially macro-aggregates^20^,^21^,^22^,^23^,^24^, which could keep SOC from decomposition by other microbial communities and thus enhance carbon storage in soil^17^,^25^,^26^. Overall, AMF are one of the vital contributors to autotrophic soil respiration by consuming carbohydrates from plants^10^,^11^. On the other hand, they may also suppress soil carbon release and promote soil carbon accumulation by protecting SOC in macro-aggregates from the activity of soil microbes^3^,^17^. Until now, the balance between these opposite effects of the fungi on soil carbon sequestration and soil respiration is still unclear^25^.

Soil respiration, which consists of autotrophic and heterotrophic parts^27^,^28^, is the largest carbon fluxes from soil^29^. Generally, soil respiration is correlated positively to the mean annual temperature without consideration of the ecosystem and biome^30^,^31^. In a single ecosystem, the inter-annual variation of soil respiration is mainly regulated by precipitation change especially in arid and semiarid ecosystems^31^,^32^. The significant influences of precipitation change on soil respiration have been confirmed in many field manipulation experiments^33^,^34^. Precipitation increase could promote soil respiration by improving plant growth^34^ and microbial activity^34^,^35^. However, the impacts of AMF on precipitation-regulated-soil respiration are still unknown.

Semiarid steppes are the most widely spread grassland ecosystems in north China. They contribute 15.8% of the world carbon storage and more than 90% of them were stored in the soil^36^. In addition, more than 80% of the plant species form symbiotic mycorrhiza with AMF in this region^37^,^38^. Thus, the ecosystem provides us a perfect platform to study the roles of AMF on soil carbon release, which are important in evaluating soil carbon balance under different precipitation regimes. In this study, a field manipulative experiment with water addition and fungicide application treatments was conducted, and soil respiration rate during three growing seasons was measured continuously to evaluate the influences of AMF on soil respiration and its response to precipitation change.

## Results

### AMF suppression

**Figure 1:**
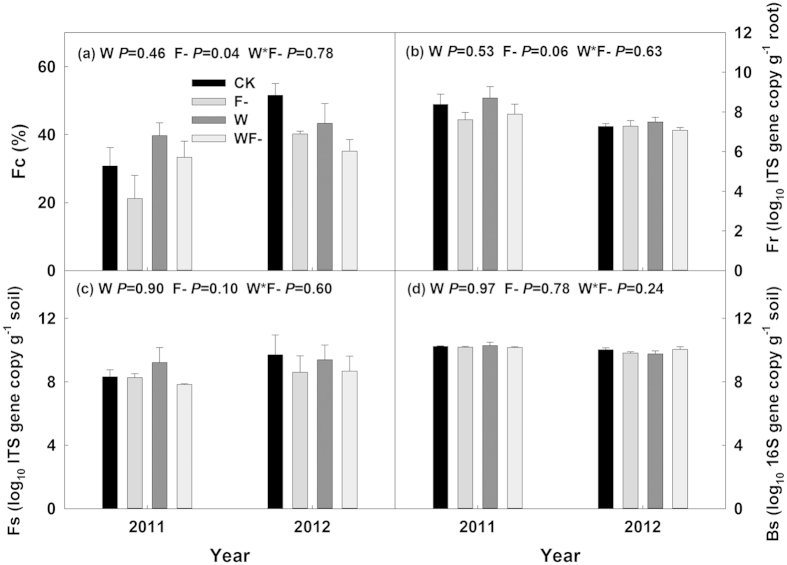
AMF root colonization (**a**, Fc), fungi in root (**b**, Fr), fungi (**c**, Fs) and bacteria (**d**, Bs) in soil under the four treatments. Results (*P*-values) of generalized linear mixed models (GLMM) were presented in each subplot. CK, control; F-, fungicide application; W, water addition; WF-, water addition plus fungicide application.

Fungicide application significantly reduced AMF root colonization by 15% (Fc; *P* = 0.04, Fig. 1a) and fungal concentration in roots by 6% (Fr; *P* = 0.06, Fig. 1b), but did not affect concentrations of fungi (Fs) and bacteria (Bs) in the soil (both *P* > 0.1, Fig. 1c,d). No significant water addition effect and its interaction with fungicide application were found on Fc, Fr, Fs and Bs (all *P* > 0.1, Fig. 1a–d).

### Precipitation patterns, soil moisture and temperature

**Figure 2:**
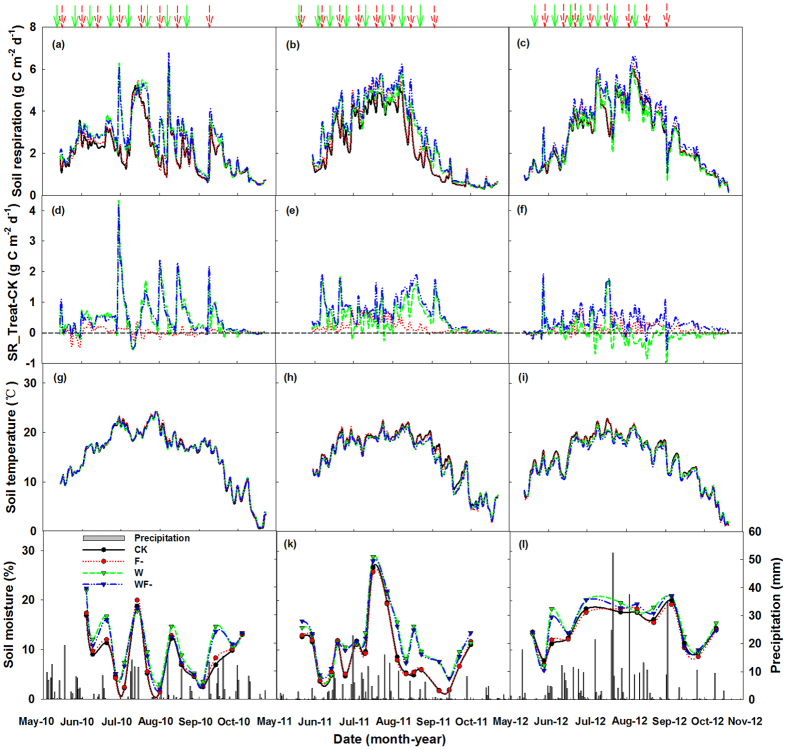
Seasonal patterns of daily soil respiration rate (**a–c**), the differences in daily soil respiration rate between the treatments and the control (**d–f**), soil temperature (**g–i**), volumetric soil moisture and precipitation (**j–l**) during the growing seasons from 2010 to 2012. Arrows above the figure showed the time when water addition (short red arrow in dash line) and fungicide (solid green arrow) were applied. CK, control plots (solid black line); F-, fungicide application (dotted line in red); W, water addition (short dash line in green); WF-, water addition plus fungicide application (dash-dot-dot line in blue).

Precipitation during the growing seasons (May to Oct) was 295 mm in 2010, 225 mm in 2011 and 434 mm in 2012 (Fig. 2j–l). Seasonal precipitation of 2010 was similar to long-term (1980–2012) average precipitation (298 mm). However, the uneven distribution of rainfall with more than 60% of the precipitation occurring at the beginning (May) and the end (September and October) of the growing season resulted in severe water deficit during peak season (June to August) (Fig. 2j). Seasonal precipitation of 2011 was 24.5% lower than long-term average; and about 81% of the precipitation occurred during the peak season (Fig. 2k), which resulted in greater soil water availability than that of 2010. Seasonal distribution of wet year 2012 was similar to 2011 with 71% precipitation occurring during the peak season (Fig. 2l). Soil moisture dynamics showed close relationships with precipitation fluctuations during the three growing seasons (Fig. 2j–l). Soil temperature showed consistent seasonal dynamics with a peak in July and August during the three growing seasons (Fig. 2g–i).

**Figure 3:**
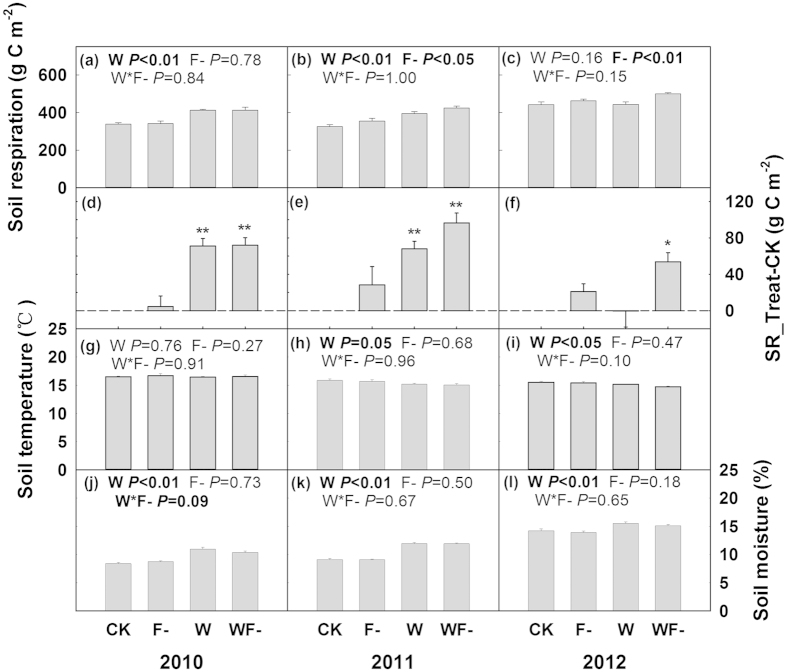
Seasonal accumulated soil respiration (**a–c**), the difference in seasonal soil respiration between the treatments and the control (**d–f**), seasonal mean soil temperature (**g–i**) and volumetric soil moisture (**j–l**) during the growing seasons from 2010 to 2012. Results (*P*-values) of two-way ANOVA were presented in each subplot (except **d–f**). * and ** on the bar (**d–f**) indicate significances from 0 at *P* < 0.05 and *P* < 0.01, respectively.

During the three growing seasons, water addition treatment decreased soil temperature by 2.7% with marginal significance (*P* = 0.07; Table 1 and Fig. 3g–i) and significantly enhanced soil moisture by 21% (*P* < 0.01; Table 1 and Fig. 3j–l). The enhancement effects were much higher in the relatively dry years. Water addition treatment enhanced soil moisture by 25% (*P* < 0.01) in 2010, by 31% (*P* < 0.01) in 2011 and 10.4% (*P* < 0.05) in 2012 (Fig. 3j–l), respectively. There was no significant variations in soil moisture or soil temperature caused by fungicide application (*P* > 0.05) and its interaction with water addition treatment (*P* > 0.05; Table 1).

### Seasonal and inter-seasonal variations of soil respiration

Soil respiration showed similar patterns during the three growing seasons, with a peak value during July-August and low rate at the beginning and end of the growing seasons (Fig. 2a–c). At the beginning and end of growing season in 2010, soil respiration was much higher compared with that in the same period of other seasons, because of higher precipitation during this period (Fig. 2a–c).

Total amounts of carbon release by soil respiration in the control plots were 299, 320 and 426 g C m^−2^ during the growing seasons in 2010–2012, respectively (Fig. 3a–c). Water addition treatment significantly improved soil respiration by 23% in 2010 and 20% in 2011 (both *P* < 0.01), while no obvious impacts in 2012 (*P* > 0.05, Fig. 3a–c). Fungicide application did not show significant impacts on soil respiration in 2010 (*P* > 0.05), while significantly promoted soil respiration by 8% in 2011 (*P* < 0.05) and by 9% in 2012 (*P* < 0.01, Fig. 2a–c). The increases of soil respiration induced by fungicide application mainly occurred on the peak seasons (Fig. 2d–f). There was no significant interaction between the effects of water addition and fungicide application on soil respiration (*P* > 0.05; Table 1 and Fig. 3a–c).

### Soil respiration components

**Figure 4:**
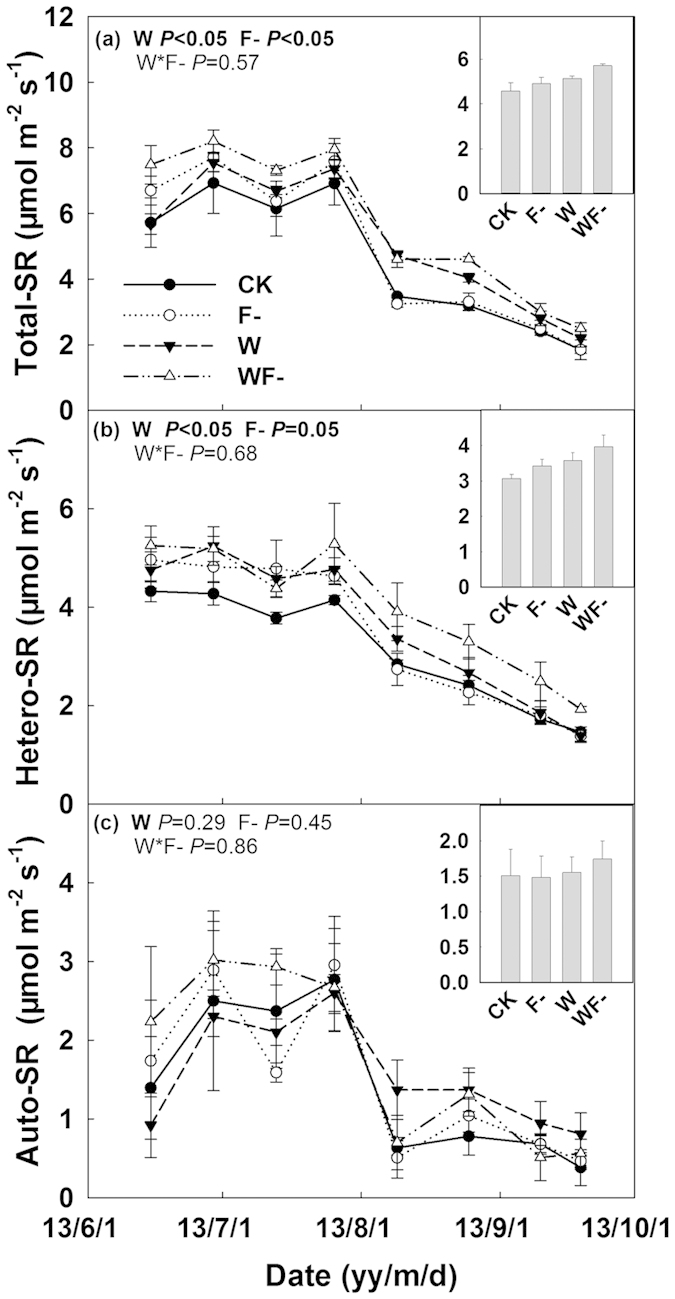
Seasonal dynamics of total soil respiration (total-SR, **a**), heterotrophic (hetero-SR, **b**) and autotrophic soil respiration (auto-SR, **c**) during the growing season in 2013. Results (*P*-values) of repeated-measures mixed model were presented in each subplot. The bar in the top right corner of each subplot indicates seasonal mean total SR, hetero-SR and auto-SR, respectively.

**Figure 5:**
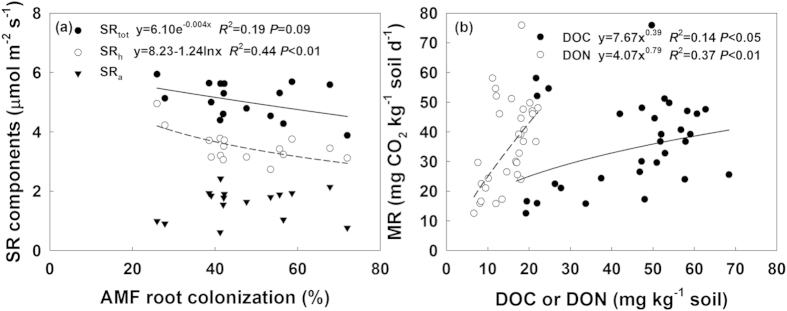
Relationships of (**a**) total-SR and its components with AMF root colonization (Fc), and (**b**) soil dissolved organic carbon (DOC) and nitrogen (DON) with microbial respiration (MR).

In 2013, seasonal mean soil respiration in the control plots was 4.6 μmol m^−2^ s^−1^, and consisted of 67% of heterotrophic and 33% of autotrophic parts (Fig. 4a–c). Both water addition and fungicide application increased soil respiration and its heterotrophic component significantly (both *P* < 0.05, Fig. 4a,b). However, the autotrophic part was not affected by both water and fungicide application treatments (both *P* > 0.05, Fig. 4c). There were no significant interactions between water addition effects and fungicide application observed on soil respiration and its components (all *P* > 0.05, Fig. 4a–c). Significantly negative relationships of soil respiration and its heterotrophic component with AMF root colonization were also found (Fig. 5a).

### Soil aggregates, dissolved organic carbon and nitrogen, microbial biomass carbon and nitrogen, microbial respiration

**Figure 6:**
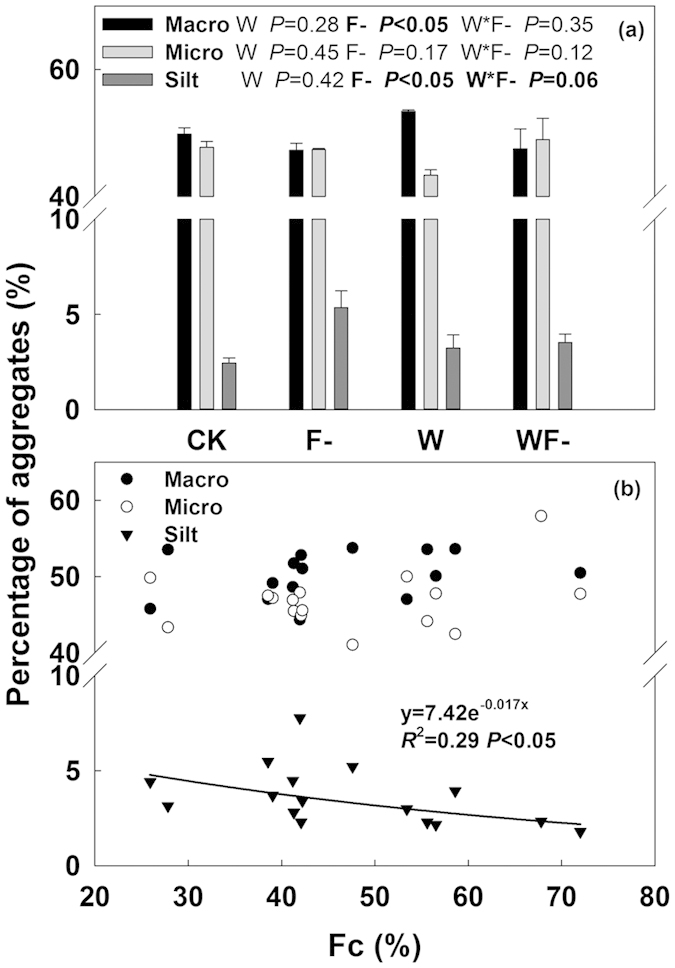
(**a**) Percentage composition of soil macro-aggregates (>250 μm in diameter), micro-aggregates (50~250 μm in diameter) and silt (<50 μm in diameter) under four treatments in 2013. Results (*P*-values) of two-way ANOVA were presented. (**b**) Relationships between AMF root colonization (Fc) and different aggregates components.

The proportion of soil macro-aggregates, micro-aggregates and silt in the control plots were 49.8%, 47.7% and 2.4%, respectively (Fig. 6a). Fungicide application significantly decreased the proportion of macro-aggregates by 8.2% and increased that of silt by 56%, respectively (both *P* < 0.05; Fig. 6a). However, there was no significant water addition effect on all aggregates composition (*P* > 0.05; Fig. 6a). A significant negative relationship was found between proportion of silt in soil and AMF root colonization (Fig. 6b).

Water addition treatment increased soil dissolved matter especially DON by 26% (*P* < 0.01; Table S1 and 1). Fungicide application also increased DON by 14% marginally significantly (*P* = 0.07; Table 1 and S1). Treatments did not cause significant changes in soil microbial biomass except an increase in MBN by 20% in water addition plots (*P* < 0.05; Table 1 and S1). In the laboratory incubation, microbial respiration (MR) from the soils of water addition and fungicide application plots was 54% (*P* < 0.01) and 26% (*P* = 0.06) higher than that of the control plots, respectively (Table 1 and S1). Significantly positive relationships of MR with DOC and DON were found, respectively (Fig. 5b).

### Responses of seasonal soil respiration to precipitation and soil moisture

**Figure 7:**
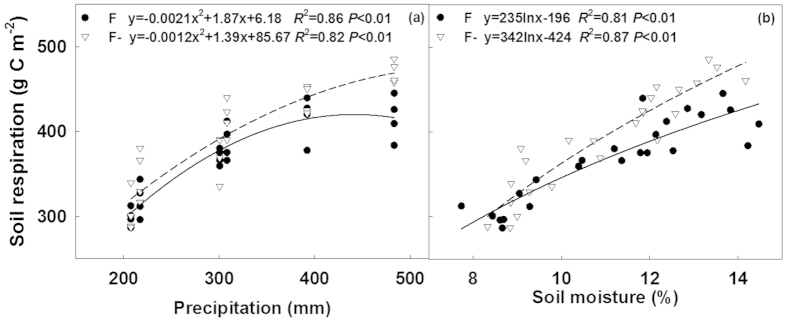
Nonlinear responses of seasonal soil respiration in the F and F- plots to precipitation (**a**) and soil moisture (**b**). F (AMF control) represented the treatments without fungicide application and included the CK and W treatments, while F- (AMF suppression) represented the treatments with fungicide application and included F- and WF- treatments.

Non-linear responses of seasonal soil respiration to precipitation were found in both AMF control plots (F, including non-fungicide CK and W treatments) and AMF suppression plots (F-, including fungicide F- and WF- treatments), respectively (Fig. 7a). In the F plots, increased rate of soil respiration showed an obvious decrease when seasonal precipitation was above 300 mm, which was close to the long-term mean precipitation (Fig. 7a). Comparatively, the change rate of soil respiration to precipitation was relatively stable in the F- plots, which resulted in significantly higher soil respiration rate with increasing precipitation than that in the F plots (Fig. 7a). Logarithmic relationships between seasonal total soil respiration and mean soil moisture were also found across the three seasons (Fig. 7b).

## Discussion

Water addition treatment significantly enhanced soil respiration except in the wet year of 2012, which induced a non-linear response of soil respiration to precipitation change and soil water content (Fig. 7a,b). Such non-linear relationship was also observed in other water manipulation experiments^33^,^34^,^39^. In semiarid ecosystems, both plant growth^40^ and microbial activity^41^ are sensitive to soil moisture, which induce a great response of soil respiration to water addition. In our study, enhanced aboveground net primary production (30%, unpublished data) and microbial respiration (54%, Table S1 and 1) by water addition were observed, and might act as the major contributors to increases in soil respiration. Enhanced microbial respiration in water addition plots was closely related to improved soil dissolved matter (especially DON). Otherwise, the sensitivity of soil respiration to water addition also depended on ambient precipitation and no significant effects of water addition were observed in the wet year of 2012 (Fig. 3l). Similar results have also been reported in studies carried out in Sonoran desert^42^ and a mesic heathland ecosystem^43^. Generally, more precipitation means more raining days and lower temperature. Immediate decreases in soil temperature and corresponding sharp declines of soil respiration rate after large rainfall (including water addition) events were observed in the experiment especially during the wet 2012, which might limit soil respiration rate and its response to water addition in further (Fig. 2f). Our results suggested that water addition would increase soil respiration, but its effects depend on the ambient water condition, which results in a nonlinear response of soil respiration to precipitation change.

Fungicide application decreased AMF colonization and fungi concentration in roots, without altering soil microbial biomass (MBC and MBN) as well as their composition (fungi and bacteria) of microbes in soil. These results were consistent to previous studies in this region^38^ and tall-grass prairies in north America^3^,^6^,^44^,^45^, and confirmed that benomyl was effective on suppressing AMF colonization in plants with minor side effects on soil microbial community.

AMF suppression treatment increased soil respiration especially under wet condition. In many greenhouse experiments, however, soil respiration in non-AMF treatment was much lower than that in AMF-inoculated treatment^46^,^47^,^48^. The presence of AMF could enhance plant productivity and thus promote soil respiration especially its autotrophic component^46^,^47^,^48^. While in the field, we did not observed significant changes in plant productivity and autotrophic soil respiration. Unchanged plant productivity has also been reported by other AMF suppression experiment^3^,^38^. In this study, enhanced soil respiration in fungicide application treatments mainly stemmed from the increase in heterotrophic component of soil respiration (Fig. 4a,b), which was proven by evidences observed in the field soil respiration separation and laboratory soil incubation experiment (Table S1 and 1 and Fig. 4). Heterotrophic soil respiration, results from soil organic carbon decomposition, was determined by both soil microbial activity and substrate availability. Previous studies have shown that AMF hyphae can promote formation of soil macro-aggregates and reduce SOC decomposition^3^,^20^,^21^,^22^,^26^. A significant decrease in soil macro-aggregate component and a remarkable increase in silt component after four-year fungicide application were observed in our experiment (Fig. 6a). The breakdown of soil macro-aggregates would promote release of soil organic matter, inducing higher soil organic matter availability (DON) and microbial activity (MR). Therefore, in spite of unchanged soil microbial biomass, the higher accessibility of substrate under fungicide application treatments triggered soil carbon release and its response to increasing precipitation, especially when precipitation was beyond the long-term average (Fig. 7).

In summary, soil respiration was triggered by water addition especially during drought season, exhibited nonlinear relationship with precipitation change. AMF symbiosis could regulate the response of soil respiration to precipitation in this Inner Mongolia steppe ecosystem. AMF suppression treatment accelerated carbon release by soil respiration up to 9% during the experiment period, which was mainly stemmed from significant increase of heterotrophic part of soil respiration. Protection of AMF on soil carbon in macro-aggregates from heterotrophic decomposition might become more important with increasing water availability. Other factors of global climate change such as elevated CO_2_^4^,^49^, nitrogen deposition^49^,^50^ and land use change^51^ have also showed to impact the species and abundance of AMF, which would eventually influence belowground carbon processes and stabilization of ecosystem carbon balance. Therefore, further studies, especially global change multiple-factor experiments, are needed to fully understand the feedback relationship between AMF with ecosystem carbon dynamic and the incorporate that into ecosystem carbon cycle researches.

## Materials and Methods

### Study site and experimental design

Our study site located at the Xilin River Basin, Inner Mongolia, China (116°42′ E, 43°38′ N, 1250 m a.s.l.). The site has been fenced since 1999 and no grazing or other disturbance thereafter. The site is a typical steppe and dominated by perennial grasses, such as *Leymus chinensis* and *Stipa grandis*, etc. Long-term (1980–2012) mean annual temperature of the study area is 0.3 °C, annual precipitation is 346 mm with more than 80% occurring during the growing season (May-October).

The experiment was carried out from early May 2010 as a two-factor random block design with water addition and fungicide application treatments. Sixteen 2 m × 2 m plots were grouped into four blocks with 1 m interval between plots. The plots were assigned randomly into four treatments in each block, including control (CK), water addition (W), fungicide application (F-) and water addition plus fungicide application (WF-). In the water addition plots, 120 mm precipitation was added with 8 times using a hand sprinkler biweekly from mid-May to early September in each season. In the fungicide treated plots, benomyl was applied to suppress AMF biweekly with 8 times but one week before the water addition. Benomyl solution (16 g benomyl dissolved in 8 L water) was applied manually to each fungicide application plot including F- and WF-, while plots of CK and W received equivalent amount of water at the same time. In this study, benomyl was chosen to suppress AMF, which is a systemic fungicide and its function only takes effect when metabolized into some anti-fungal catabolites *in vivo* of plants. Previous studies have reported its efficient in reducing AMF infection in roots but with fewest side effects on soil directly^3^,^6^,^38^,^44^,^45^. In order to minimize edge effects, all the measurements and samplings were conducted in the central zone with a 20 cm buffer zone around the plots.

### Continuous soil respiration and environmental factors measurements

Soil respiration and environmental factors were measured continuously during the growing seasons of 2010–2012. At the beginning of the experiment, one PVC collar (20.3 cm in diameter and 8 cm in height) was inserted into the soil to a depth of 3 cm in each plot. Any live plants inside the collars were removed immediately once found. A continuous soil respiration measurement system was installed at middle May of 2010. The system consists of 16 automatic chambers (Truwel Inc., Beijing, China) attached to a multiplexer (Truwel Inc.), an infrared gas analyzer (LI-840, LI-COR Inc., Lincoln, USA), an air pump (LI-COR Inc.), and a power supply system (Dahe Inc., Beijing, China). Soil respiration rate in each plot was measured every two hours during the growing seasons. Each measurement lasted 150 s including 30 s for the closing and opening of the chamber. Concentrations of CO_2_, H_2_O and air temperature inside the chamber were measured every 2 s and recorded using CR5000 data-logger (Campbell Scientific Inc., Logan, IL, USA). Soil respiration rates were calculated based on the slope of the linear regression of CO_2_ concentration with time.

Soil temperature at a depth of 10 cm was also continuously measured every 2-hours by type E thermocouple (REOTEMP Inc., San Diego, USA) and recorded in CR5000 data-logger. Volumetric soil moisture of 0–10 cm was measured weekly using a portable TDR-200 soil moisture probe (Spectrum Technologies Inc., Plainfield, USA). Precipitation data were recorded in a weather station about 300 m away from the experiment site.

### Separation of soil respiration components

A supplementary experiment by mini-trenching method^32^,^52^ was conducted to separate soil respiration in 2013. Briefly, a shallow PVC collar (10 cm in diameter and 6 cm in depth) and a deep collar (10 cm in diameter and 40 cm in depth) were inserted into the soil in each plot both with 3 cm above the ground in April 2013. Soil respiration measured from the two collars could be represented as total and heterotrophic soil respiration. Their difference was the autotrophic part^32^,^49^. Soil respiration in this season was measured biweekly from early May to September using a portable LI-8100 automated soil flux system (LI-COR Inc.).

Like other soil respiration partitioning methods used in grasslands such as clipping and trenching, the mini-trenching method may also overestimate heterotrophic soil respiration for the dead root decomposition or underestimate it due to the less roots exudates^27^,^28^,^32^. However, compared with other partitioning approaches^27^,^28^, this method is simple, economical and space efficient^32^, and meets the goal of our experiment. In order to avoid disturbances caused by collar installation, only data after two month of the installation were used in analysis, when the constituents of soil respiration tend to be steady with about 70% as heterotrophic soil respiration.

### Soil microbial biomass, microbial respiration and dissolved organic matter

In August 2012 and 2013, three soil core samples (10 cm in depth and 7 cm in diameter) were collected from each plot and mixed into one sample for the laboratorial analyses. Soil microbial biomass carbon (MBC) and nitrogen (MBN) were determined using fumigation-extraction method^41^,^53^. Soil dissolved organic carbon (DOC) and nitrogen (DON) were estimated by measuring the extractable carbon and nitrogen contents in the unfumigated samples^41^,^53^. Microbial respiration (MR) was determined by measuring alkali absorption of CO_2_ emitted at 25 ^o^C for 4 days followed by titrating the remaining OH^−^ with a standard acid^41^.

### AMF root colonization

In the peak seasons, three soil cores (10 cm in depth and 7 cm in diameter) were collected from each plot, and live roots were collected and mixed into one sample. The root samples were cleared in 10% KOH at 90 °C in a water bath for 40 min. After washed and drown into 2% HCL solution for 5 min, roots were stained in acid fuchsin-Lactic acid-Glycerin stain solution at 90 °C for 20 min. 25 root segments with length of 20 mm were examined microscopically to assess the percentage of the root length colonized by AMF (Fc)^54^.

### Root fungi, soil fungi and bacteria

Total roots genomic DNA were extracted from 100 mg of roots using the DNeasy plant DNA extraction kit (Tiangen Biotech CO., Beijing, China) to examine fungi in roots (Fr). Total soil genomic DNA was also extracted from 250 mg of soil samples using the MoBio Soil DNA isolation kit (MoBio Laboratories, Inc., Carlsbad, USA) to determine fungal (Fs) and bacterial (Bs) concentration in soil. Both the DNA was eluted twice in 50 μL of buffer TE (10 mM Tris-HCl, 1 mM EDTA, pH = 8.0) and was stored at −20 °C until real-time PCR amplification.

Real-time PCR assays were conducted using StepOnePlusTM Instrument (Applied Biosystems). Each 20 μL of the amplification reaction solution contained the following reagents: 10 μL of SYBR Premix Ex Taq (Takara Bio Inc., Dalian, China), 0.4 μL of each primer (10 mmol mL^−1^), 0.4 μL of ROX Reference Dye (50 × ), 6.8 μL of H_2_O, and 2 μL of template DNA. The primer pair ITS1F and ITS4 was adopted in PCR for all fungal groups^55^, the primer pair 27F and 533R was used for PCR amplification of all bacteria group^56^. The PCR testing followed the procedures of 30 s at 95 °C, 40 cycles at 95 °C for 10 s, 30 s at 56 °C for annealing, 72 °C for 1 min, and 80 °C for 10 s. The concentrations were all represented by the logarithm of gene copy number per gram roots or soil.

### Soil aggregates

Water-stable aggregates were separated into four different size classes (>2000, 2000~250, 250~50 and <50 μm in diameter) using wet-sieving method^57^, which involved three nested sieves of 2000 μm, 250 μm and 50 μm in pore size, respectively. The <50 μm and 50~250 μm size classes were referred to as silt and micro-aggregates, respectively; while the 250~2000 and >2000 μm size classes were referred to as macro-aggregates^57^.

### Statistical analysis

The original and derived variables were analyzed with generalized linear mixed models (GLMM) to test the treatment effects on AMF root colonization (Fc), fungi in root (Fr), fungal concentration in soil (Fs), bacterial concentration in soil (Bs), seasonal soil respiration (SR), soil temperature (ST) and moisture (SM), soil dissolved organic carbon (DOC) and nitrogen (DON), microbial biomass carbon (MBC) and nitrogen (MBN) and microbial respiration (MR). Water addition, fungicide treatment, year and their interactions were considered as fixed effects; block was considered as a random effect^58^. These analyses were performed using ASReml 3.0 (VSN International Ltd., Hemel Hempstead, U.K.). Two-way ANOVA were conducted to test treatment effects on soil aggregates in 2013. Repeated-measures mixed model were conducted to test the water addition and fungicide application effects on SR components. Regression analysis was conducted to determine the relationships of Fc with soil aggregates and SR components, inter-annual precipitation with SR, seasonal mean SM with SR, DOC and DON with MR, respectively. These statistical analyses were performed using SAS 9.1 (SAS Institute Inc., Cary, USA).

## Additional Information

**How to cite this article**: Zhang, B. *et al*. Arbuscular mycorrhizal fungi regulate soil respiration and its response to precipitation change in a semiarid steppe. *Sci. Rep*. **6**, 19990; doi: 10.1038/srep19990 (2016).

## Supplementary Material

Supplementary Information

## Figures and Tables

**Table 1:** P-values of generalized linear mixed models (GLMM) showing the effects of block (random factor), water addition (W), fungicide application (F-), year (Y), and their interactions on soil dissolved organic carbon (DOC, mg kg^−1^ soil) and nitrogen (DON, mg kg^−1^ soil), soil microbial biomass carbon (MBC, mg kg^−1^ soil) and nitrogen (MBN, mg kg^−1^ soil) and microbial respiration (MR, mg CO_2_ kg^−1^ soil day^−1^), seasonal mean soil temperature (ST, °C) at 10 cm soil depth and soil moisture (SM, %) of the 0–10 cm soil layer and seasonal total soil respiration (SR, g C m^−2^).

Source	DOC	DON	MBC	MBN	MR	ST	SM	SR
Block	0.564	0.695	0.207	0.265	0.497	0.340	0.296	0.086
W	0.120	0.006	0.348	0.048	0.003	0.070	<0.001	<0.001
F-	0.137	0.074	0.991	0.626	0.059	0.290	0.222	0.013
W × F-	0.593	0.562	0.460	0.685	0.326	0.436	0.152	0.536
Y	<0.001	<0.001	<0.001	<0.001	0.011	<0.001	<0.001	<0.001
Y × W	0.694	0.292	0.905	0.445	<0.001	0.115	<0.001	<0.001
Y × F-	0.018	0.455	0.097	0.064	0.077	0.865	0.474	0.002
Y × W × F-	0.182	0.590	0.205	0.059	0.584	0.355	0.263	0.098
