# Supplementary material for: Arbuscular mycorrhizal fungi regulate soil respiration and its response to precipitation change in a semiarid steppe
Source: Sci Rep. 2016 Jan 28;6:19990. doi: 10.1038/srep19990 (PMC4730203; doi:10.1038/srep19990)
Supplement: Supplementary Information [file srep19990-s1.doc]

**Title:** Arbuscular mycorrhizal fungi regulate soil respiration and its response to precipitation change in a semiarid steppe

**Authors:** Bingwei Zhang^1,2^, Shan Li^1,2^, Shiping Chen^1*^, Tingting Ren^1^, Zhiqiang Yang^1,2^, Hanlin Zhao^1^, Yu Liang^1*^, Xingguo Han^1,3^

1 State Key Laboratory of Vegetation and Environmental Change, Institute of Botany, Chinese Academy of Sciences, Beijing, 100093, China

2 University of Chinese Academy of Sciences, Beijing, 100049, China

3 State Key Laboratory of Forest and Soil Ecology, Institute of Applied Ecology, Chinese Academy of Sciences, Shenyang 110164, China

***Corresponding author:** Shiping Chen,

20 Nanxincun, Xiangshan,

Beijing 100093, China

Tel: 86-10-62836233, Fax: 86-10-62596874

E-mail: spchen@ibcas.ac.cn

**Yu Liang**

20 Nanxincun, Xiangshan,

Beijing 100093, China

Tel: 86-10-62836958

E-mail: coolrain@ibcas.ac.cn

Table S1 Soil dissolved organic carbon (DOC, mg kg^-1^ soil) and nitrogen (DON, mg kg^-1^ soil), soil microbial biomass carbon (MBC, mg kg^-1^ soil) and nitrogen (MBN, mg kg^-1^ soil) and microbial respiration (MR, mg CO_2_ kg^-1^ soil day^-1^) under four treatments during the growing seasons of 2012 and 2013. CK, control; F-, fungicide application; W, water addition; WF-, water addition plus fungicide application.

|  | Treatment | DOC | DON | MBC | MBN | MR |
| --- | --- | --- | --- | --- | --- | --- |
| 2012 | CK | 49.5±1.5 | 16.5±0.7 | 443±27 | 68.3±3.9 | 29.7±1.3 |
|  | F- | 54.2±1.8 | 17.7±0.7 | 426±36 | 64.2±5.9 | 48±10.9 |
|  | W | 62±2.4 | 18.8±1 | 432±38 | 68.3±6.9 | 37.3±4.5 |
|  | WF- | 56.2±2 | 20.1±0.5 | 498±35 | 79.6±5.8 | 44.9±2.8 |
| 2013 | CK | 23.6±3.4 | 8.8±1.1 | 383±18 | 33.1±1.4 | 15.2±0.9 |
|  | F- | 34.9±5 | 10.4±1.1 | 354±34 | 29.3±3.7 | 21.3±1.5 |
|  | W | 27.7±7.6 | 12.2±2.2 | 406±49 | 49.3±8.4 | 46.1±6.1 |
|  | WF- | 41.5±6 | 16.1±2.4 | 385±43 | 37.2±6 | 47±3.2 |
